# Comprehensive characterization of motor and coordination functions in three adolescent wild-type mouse strains

**DOI:** 10.1038/s41598-021-85858-3

**Published:** 2021-03-22

**Authors:** Ahmed Eltokhi, Barbara Kurpiers, Claudia Pitzer

**Affiliations:** 1 Department of Neurology and Epileptology, Hertie Institute for Clinical Brain Research, University of Tübingen, Tübingen, Germany; 2 Interdisciplinary Neurobehavioral Core, Heidelberg University, Heidelberg, Germany; 3 Present Address: Department of Pharmacology, University of Washington, Seattle, USA

**Keywords:** Neuroscience, Motor control

## Abstract

Neuropsychiatric disorders are often associated with motor and coordination abnormalities that have important implications on the etiology, pathophysiology, and management of these disorders. Although the onset of many neuropsychiatric disorders including autism spectrum disorder, schizophrenia, and attention-deficit hyperactivity disorder emerges mainly during infancy and adolescence, most of the behavioral studies in mice modeling neuropsychiatric phenotypes are performed in adult animals, possibly missing valuable phenotypic information related to the effect of synaptic maturation during development. Here, we examined which behavioral tests assessing both motor and coordination functions can be performed in mice at two different adolescent stages. As strain and sex affect mouse behavior, our experiments covered both male and female mice of three inbred wild-type strains, C57BL/6N, DBA/2, and FVB/N. Adolescent mice of both postnatal days (P)22–30 and P32–40 developmental stages were capable of mastering common motor and coordination tests. However, results differed significantly between strains and sexes. Moreover, the 10-day interval between the two tested cohorts uncovered a strong difference in the behavioral results, confirming the significant impact of maturation on behavioral patterns. Interestingly, the results of distinct behavioral experiments were directly correlated with the weight of mice, which may explain the lack of reproducibility of some behavioral results in genetically-modified mice. Our study paves the way for better reproducibility of behavioral tests by addressing the effect of the developmental stage, strain, sex, and weight of mice on achieving the face validity of neuropsychiatric disorder-associated motor dysfunctions.

## Introduction

For a long time, deficits in motor function in neuropsychiatric disorders have been traditionally neglected both in clinical practice and research despite their clinical and neurobiological relevance. Fortunately, they are now considered main diagnostic criteria of many neuropsychiatric disorders^1^. For instance, coordination and motor dysfunctions have been well documented in autism spectrum disorder (ASD)^2–6^, schizophrenia^7–11^, attention-deficit hyperactivity disorder (ADHD)^12–14^, and depression^15–17^ and have important implications for the etiology^18,19^, pathophysiology^20^, and management^21,22^ of these disorders. Moreover, they are considered established markers of illness episodes and severity^19^. These motor abnormalities vary greatly in different neuropsychiatric disorders. ASD patients are characterized by poor visual tracking, limited gross and fine motor functions when grasping or reaching for objects, inability to execute a sequence of actions (apraxia and difficulties in imitation), and postural balance difficulties^23,24^. Another class of motor-related alterations is observed in schizophrenia patients which include psychomotor slowing^25,26^, poor performance in complex motor tasks^27,28^, dystonia, akathisia, and hypokinesia^27,29^. On the other hand, 30% to 50% of children with ADHD show impaired gross motor and fine motor skills including difficulties in handwriting and clumsiness in performing motor skills, which can be attributed to their hyperactivity, impulsivity, and inattention^13,30,31^.

The onset of several neuropsychiatric disorders emerges mainly during infancy and adolescence^32^. Thus, 50% of adults with neuropsychiatric disorders received a diagnosis before 15 years of age^33^. Motor abnormalities in ASD and ADHD specifically appear within the first year of life^30,34–38^, and it is known that neuromotor dysfunction in schizophrenia is even present before the onset of the disease and constitute an important indicator of it^39,40^, with poor coordination, clumsiness, and unfamiliar movements being the most common motor abnormalities among children later on developing schizophrenia^41^. However, behavioral studies modeling neuropsychiatric disorders in genetically-engineered mouse lines are mostly performed in adulthood taking the advantages of easier handling and the capabilities of mastering more complex behavioral and cognitive tasks. Unfortunately, these studies miss valuable information on the impact of synaptic maturation during development. Yet, our previous studies unraveled the capacity of adolescent mice in performing experiments assessing neuropsychiatric-like phenotypes including communication deficits^42^, anxiety, social impairment, and cognitive dysfunction^43^.

In the present study, we aimed to investigate which behavioral tests recapitulating neuropsychiatric disorder-associated motor abnormalities can be performed in adolescent mice till P40. We have selected behavioral tests with high face validity that are commonly used to evaluate traits in sensory-motor functions. Our test battery included the grip strength, beam balance rod, inverted screen, cliff avoidance reaction, rotarod, and voluntary wheel running tests. As we have previously shown that behavioral results including ultrasonic vocalizations (USV)^42^ and home-cage activity^43^ are sensitive to a small developmental progress during adolescence, we performed our behavioral test battery on two cohorts of mice, one from P22 to P30 and the other from P32 to P40 in order to draw a full picture of the motor ability during adolescence. Because the genetic background and sexes are known to influence behavioral characteristics^42–45^, we performed our experiments on male and female mice of three different inbred strains, C57BL/6N, DBA/2, and FVB/N that are world-wide used in research and are the standard strains in neuroscience with their ability to discern the role of individual genes and the impact of allelic variation along with decreased variability^46–49^.

Our work revealed that the motor activity and coordination functions during adolescence differ among these mouse strains, highlighting the potential advantages and disadvantages of individual strains in behavioral studies.

## Results

### Motor activity and coordination function in P22–30 cohort

**Figure 1:**
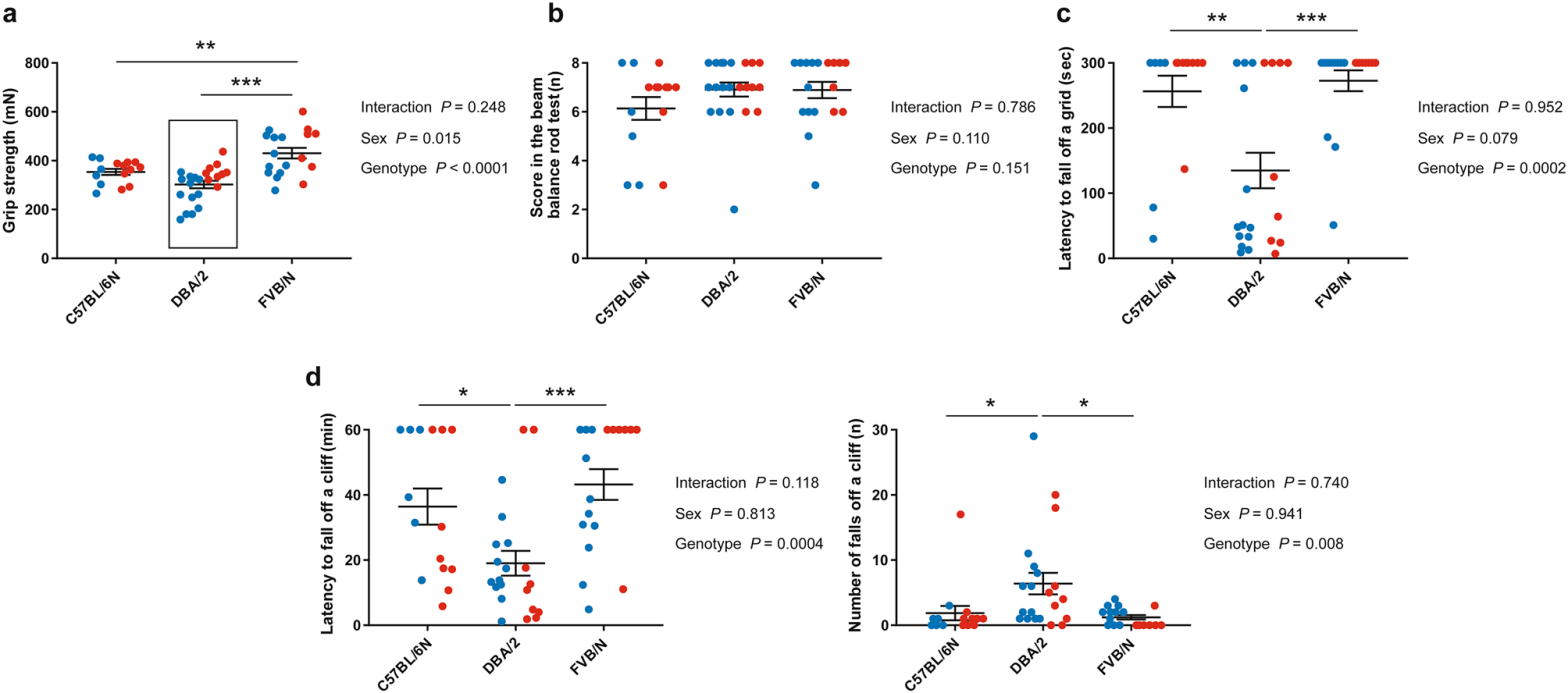
Coordination function in the P22–30 cohort of C57BL/6N, DBA/2, and FVB/N strains. (**a**) The grip strength test revealed increased grip strength for FVB/N mice compared to C57BL/6N and DBA/2 mice. (**b**) The beam balance rod test showed no difference between the three investigated strains. (**c**) DBA/2 mice displayed the lowest latency to fall off the grid in the inverted screen test. (**d**) In the cliff avoidance reaction test, DBA/2 mice exhibited the lowest latency to fall off the cliff (left) and the highest number of falls (right) compared to C57BL/6N and FVB/N mice. Two-way ANOVA followed by Tukey post hoc test, *p ≤ 0.05, **p ≤ 0.01, ***p ≤ 0.001. A black rectangle indicates a significant difference between sexes within a strain (see Supplementary Table 2). Blue and red dots refer to males and females, respectively. Error bars indicate the standard error of the mean (SEM).

The behavioral test battery started with measuring the grip strength of P22 mice. FVB/N mice revealed the most powerful grip strength compared to C57BL/6N (*P* = 0.007) and DBA/2 (*P* < 0.0001) mice (Fig. 1a). Notably, female DBA/2 mice showed a higher grip strength than males (Supplementary Table 2). For measuring coordination functions, we employed several tests. In the beam balance rod test at P23, no difference was found between the three investigated strains with a similar performance of male and female mice within each strain (Fig. 1b). In contrast, DBA/2 mice displayed the least coordination function in the inverted screen test by showing the lowest latency to fall off a grid compared to C57BL/6N (*P* = 0.002) and FVB/N (*P* = 0.0003) mice (Fig. 1c). In another test for measuring coordination and impulsivity, the cliff avoidance reaction test, DBA/2 mice revealed also the lowest latency to fall off a cliff (*P* = 0.026 vs. C57BL/6N, *P* = 0.0008 vs. FVB/N) and the highest number of falls (*P* = 0.046 vs. C57BL/6N, *P* = 0.013 vs. FVB/N) (Fig. 1d). No difference in the coordination function between male and female mice of the investigated strains was seen in either the inverted screen or cliff avoidance reaction tests (Supplementary Table 2).

**Figure 2:**
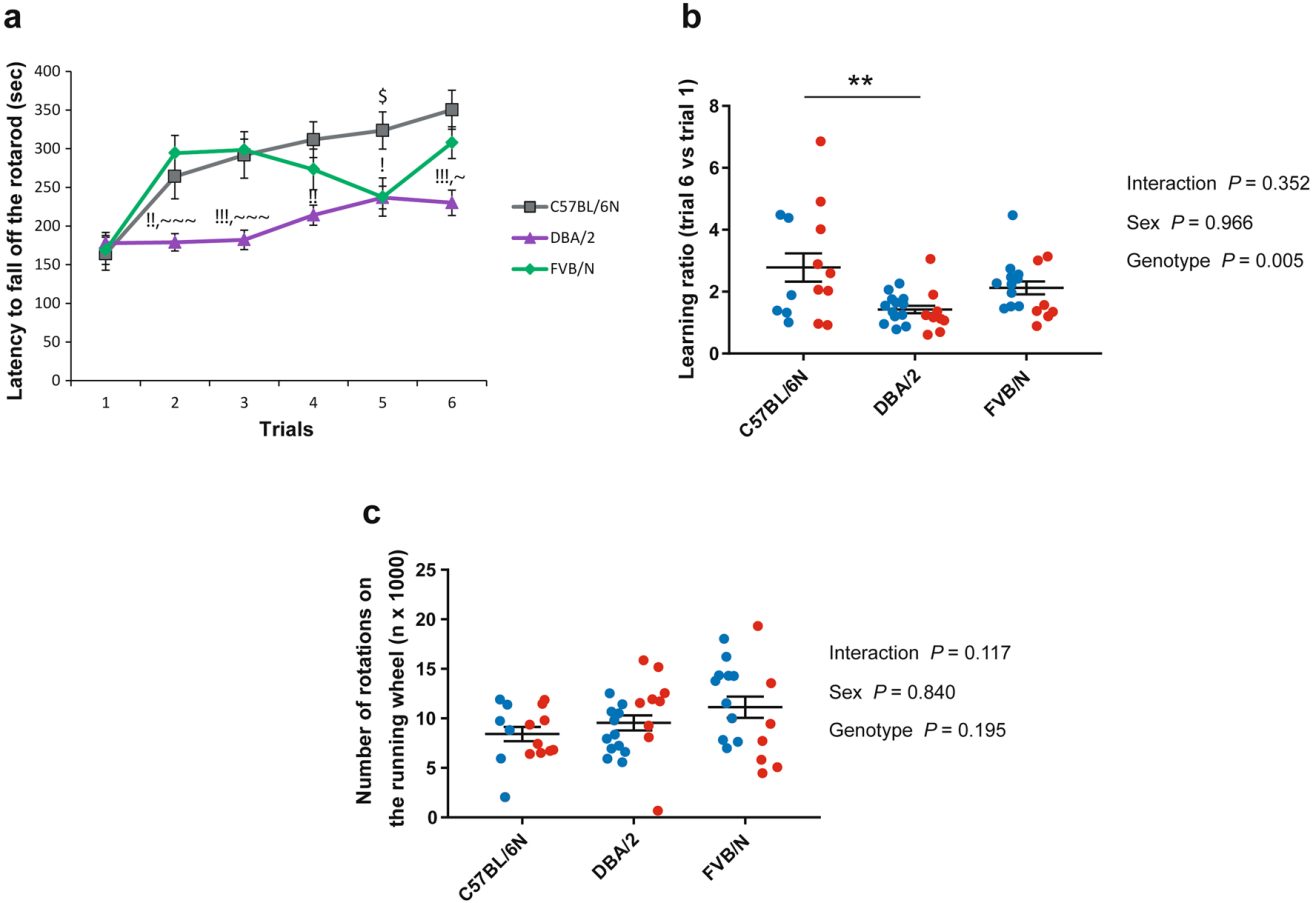
Motor activity in the P22–30 cohort of C57BL/6N, DBA/2, and FVB/N strains. (**a**) In the rotarod test, DBA/2 mice exhibited the lowest duration on the rotarod compared to C57BL/6N and FVB/N mice. For the detailed comparison between male and female mice within each strain, see Supplementary Fig. 2. (**b**) C57BL/6N mice showed a significant better learning ability in the rotarod test compared to DBA/2 mice. (**c**) In the voluntary wheel running test, no difference between the three investigated strains was revealed. Two-way ANOVA followed by Tukey post hoc test, !*p* ≤ 0.05, !!*p* ≤ 0.01 and !!!*p* ≤ 0.001 for DBA/2 versus C57BL/6N; ~ *p* ≤ 0.05 and ~~~ *p* ≤ 0.001 for DBA/2 versus FVB/N; $ *p* ≤ 0.001 C57BL/6N versus FVB/N; **p ≤ 0.01. Blue and red dots refer to males and females, respectively. Error bars indicate the standard error of the mean (SEM).

For testing the motor activity of adolescent mice, we employed two tests, the rotarod and voluntary wheel running. In the rotarod test, DBA/2 mice displayed the lowest ability to perform the test by showing a decreased latency to fall off the accelerating rotarod, which reached significance compared to C57BL/6N in all trials except trial 1 that acted as a habituation trial (Trial 1: *P* = 0.846 vs. C57BL/6N, *P* = 0.924 vs. FVB/N; Trial 2: *P* = 0.007 vs. C57BL/6N, *P* = 0.0001 vs. FVB/N; Trial 3: *P* = 0.0001 vs. C57BL/6N, *P* < 0.0001 vs. FVB/N; Trial 4: *P* = 0.003 vs. C57BL/6N, *P* = 0.073 vs. FVB/N; Trial 5: *P* = 0.012 vs. C57BL/6N, *P* = 0.999 vs. FVB/N; Trial 6: *P* = 0.0005 vs. C57BL/6N, *P* = 0.02 vs. FVB/N) (Fig. 2a). Interestingly, female C57BL/6N mice showed a better motor function in the rotarod test compared to males in trials 2, 3, and 4 (Supplementary Fig. 2; Supplementary Table 2). To check the ability of mice to learn the test, the performance of each mouse in trial 6 was compared to trial 1. C57BL/6N mice revealed the highest motor learning ability and reached significance compared to DBA/2 mice (*P* = 0.002 vs. DBA/2, *P* = 0.219 vs. FVB/N) (Fig. 2b). On the other hand, the activity of the three investigated strains in the voluntary wheel running test was comparable with no difference between male and female mice within each strain (Fig. 2c; Supplementary Table 2).

### Motor activity and coordination function in P32–40 cohort

**Figure 3:**
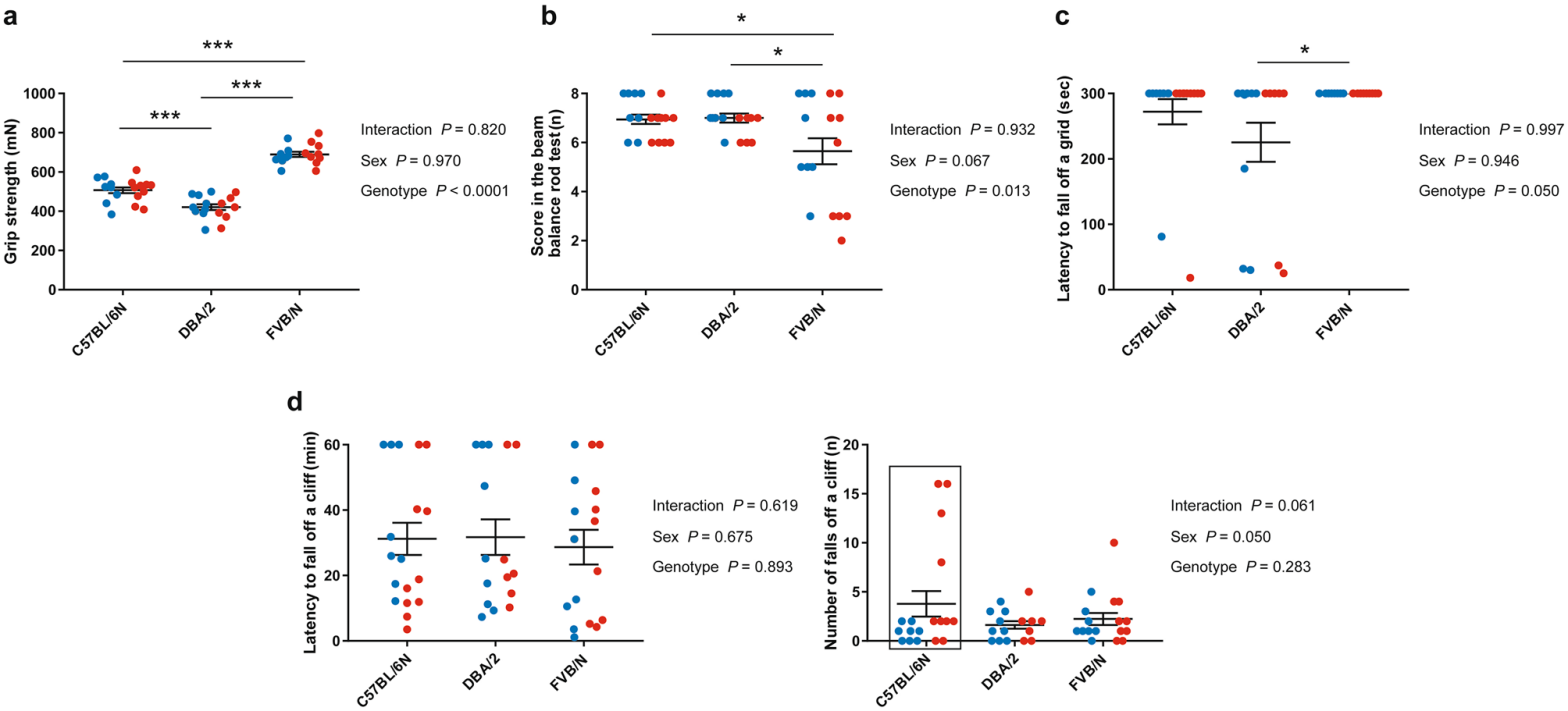
Coordination function in the P32–40 cohort of C57BL/6N, DBA/2, and FVB/N strains. (**a**) The grip strength test revealed FVB/N displaying the most powerful grip strength, DBA/2 showing the least grip strength with C57BL/6N being intermediate. (**b**) FVB/N displayed the lowest score in the beam balance rod test compared to C57BL/6N and DBA/2. (**c**) DBA/2 mice had the lowest latency to fall off a grid in the inverted screen test and reached significance compared to FVB/N mice. (**d**) In the cliff avoidance reaction test, no difference in the latency to fall off a cliff (left) or the number of falls (right) between the three investigated strains. Two-way ANOVA followed by Tukey post hoc test, *p ≤ 0.05, ***p ≤ 0.001. A black rectangle indicates a significant difference between sexes within a strain (see Supplementary Table 2). Blue and red dots refer to males and females, respectively. Error bars indicate the standard error of the mean (SEM).

The grip strength test at P32 revealed that FVB/N mice displayed the most powerful grip strength (*P* < 0.0001 vs. C57BL/6N and DBA/2) with DBA/2 mice showing the least grip strength (*P* = 0.0003 vs. C57BL/6N) and C57BL/6N mice being intermediate (Fig. 3a). Compared to the grip strength test performed at P22, the three investigated strains at P32 showed no difference between male and female mice (Supplementary Table 2). In the beam balance rod test, FVB/N mice showed a lower score compared to C57BL/6N (*P* = 0.023) and DBA/2 (*P* = 0.021) mice (Fig. 3b). In the inverted screen test, DBA/2 mice showed the lowest latency to fall off the grid but reached significance only compared to FVB/N (*P* = 0.041) (Fig. 3c). In both beam balance rod and inverted screen tests, no difference between male and female mice within strain was found (Supplementary Table 2). In the cliff avoidance reaction test, the three investigated strains exhibited comparable coordination function with no difference in the latency or number of falls off the cliff (Fig. 3d). In contrast to the cliff avoidance reaction test performed at P28 which showed no difference between male and female mice, P38 C57BL/6N female mice showed a higher number of falls compared to males (Supplementary Table 2).

**Figure 4:**
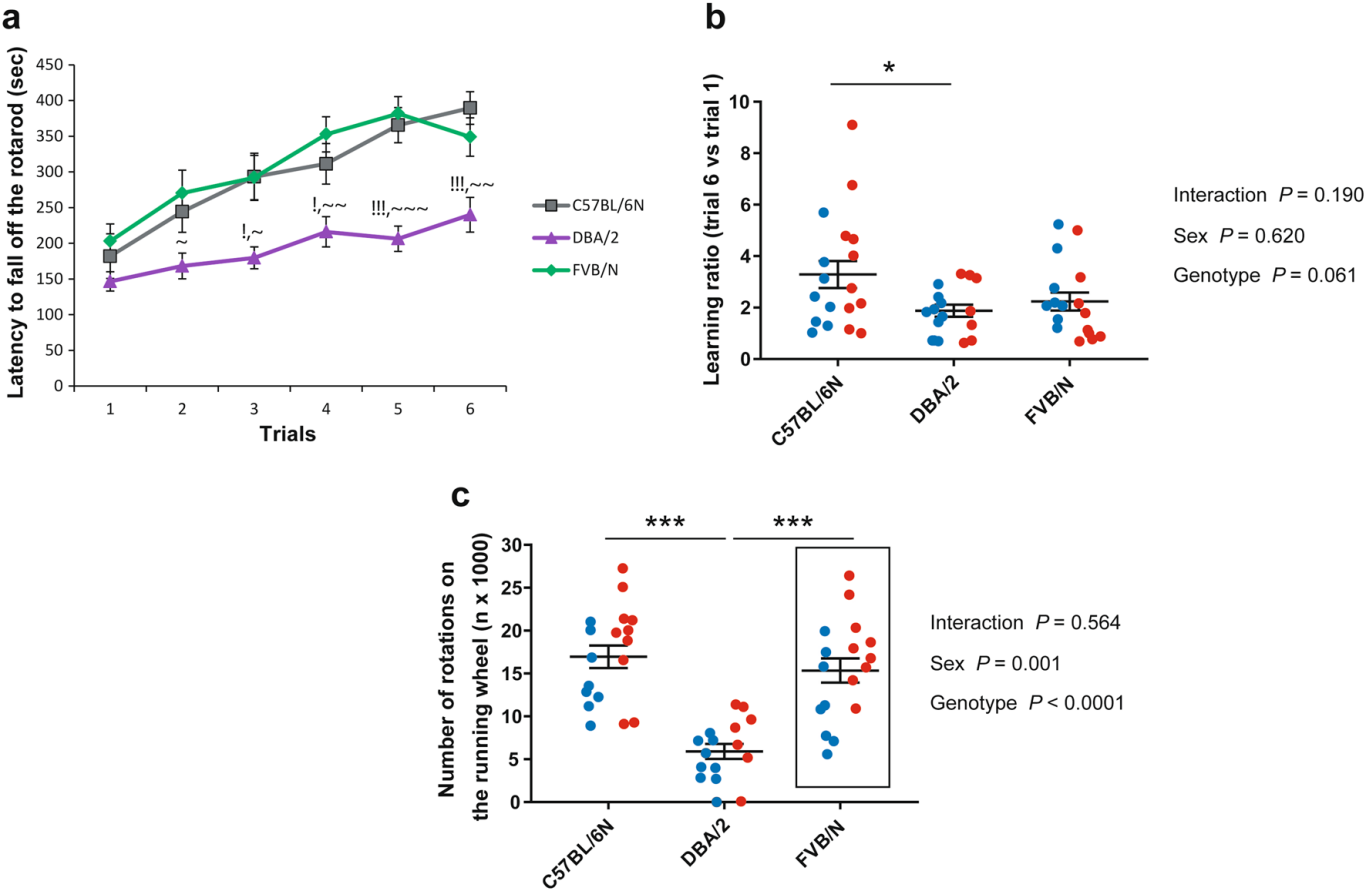
Motor activity in the P32–40 cohort of C57BL/6N, DBA/2, and FVB/N strains. (**a**) In the rotarod test, DBA/2 mice exhibited the lowest duration on the rotarod compared to C57BL/6N and FVB/N mice. For the detailed comparison between male and female mice within each strain, see Supplementary Fig. 3. (**b**) C57BL/6N mice showed a significant better learning ability in the rotarod test compared to DBA/2 mice. **C)** In the voluntary wheel running test, DBA/2 mice displayed the lowest number of rotations. Two-way ANOVA followed by Tukey post hoc test, !*p* ≤ 0.05 and !!!*p* ≤ 0.001 for DBA/2 versus C57BL/6N; ~ *p* ≤ 0.05, ~~ *p* ≤ 0.01 and ~~~ *p* ≤ 0.001 for DBA/2 versus FVB/N; *p ≤ 0.05, ***p ≤ 0.001. A black rectangle indicates a significant difference between sexes within a strain (see Supplementary Table 2). Blue and red dots refer to males and females, respectively. Error bars indicate the standard error of the mean (SEM).

Similar to the rotarod test performed at P26–27, P36–37 DBA/2 mice displayed the worst motor activity by showing a decreased latency to fall off the rotarod, which reached significance compared to FVB/N in all trials except trial 1 (Trial 1: *P* = 0.591 vs. C57BL/6N, *P* = 0.28 vs. FVB/N; Trial 2: *P* = 0.121 vs. C57BL/6N, *P* = 0.028 vs. FVB/N; Trial 3: *P* = 0.012 vs. C57BL/6N, *P* = 0.014 vs. FVB/N; Trial 4: *P* = 0.027 vs. C57BL/6N, *P* = 0.001 vs. FVB/N; Trial 5: *P* < 0.0001 vs. C57BL/6N, *P* < 0.0001 vs. FVB/N; Trial 6: *P* < 0.0001 vs. C57BL/6N, *P* = 0.005 vs. FVB/N) (Fig. 4a). Interestingly, female C57BL/6N mice showed better motor function in the rotarod test compared to males with borderline significance in trial 2 and significant difference in trial 3 (Supplementary Fig. 3; Supplementary Table 2). For motor learning, C57BL/6N mice showed the highest ability to learn the test and reached significance compared to DBA/2 mice (*P* = 0.041 vs. DBA/2, *P* = 0.149 vs. FVB/N) (Fig. 4b). Similar to the rotarod test, DBA/2 mice displayed the lowest number of rotations in the voluntary wheel running test compared to C57Bl/6N and FVB/N (*P* < 0.0001) mice (Fig. 4c). Additionally, FVB/N female mice showed an increase in the number of rotations compared to males (Supplementary Table 2).

### Comparison between the two developmental stages within each strain

**Figure 5:**
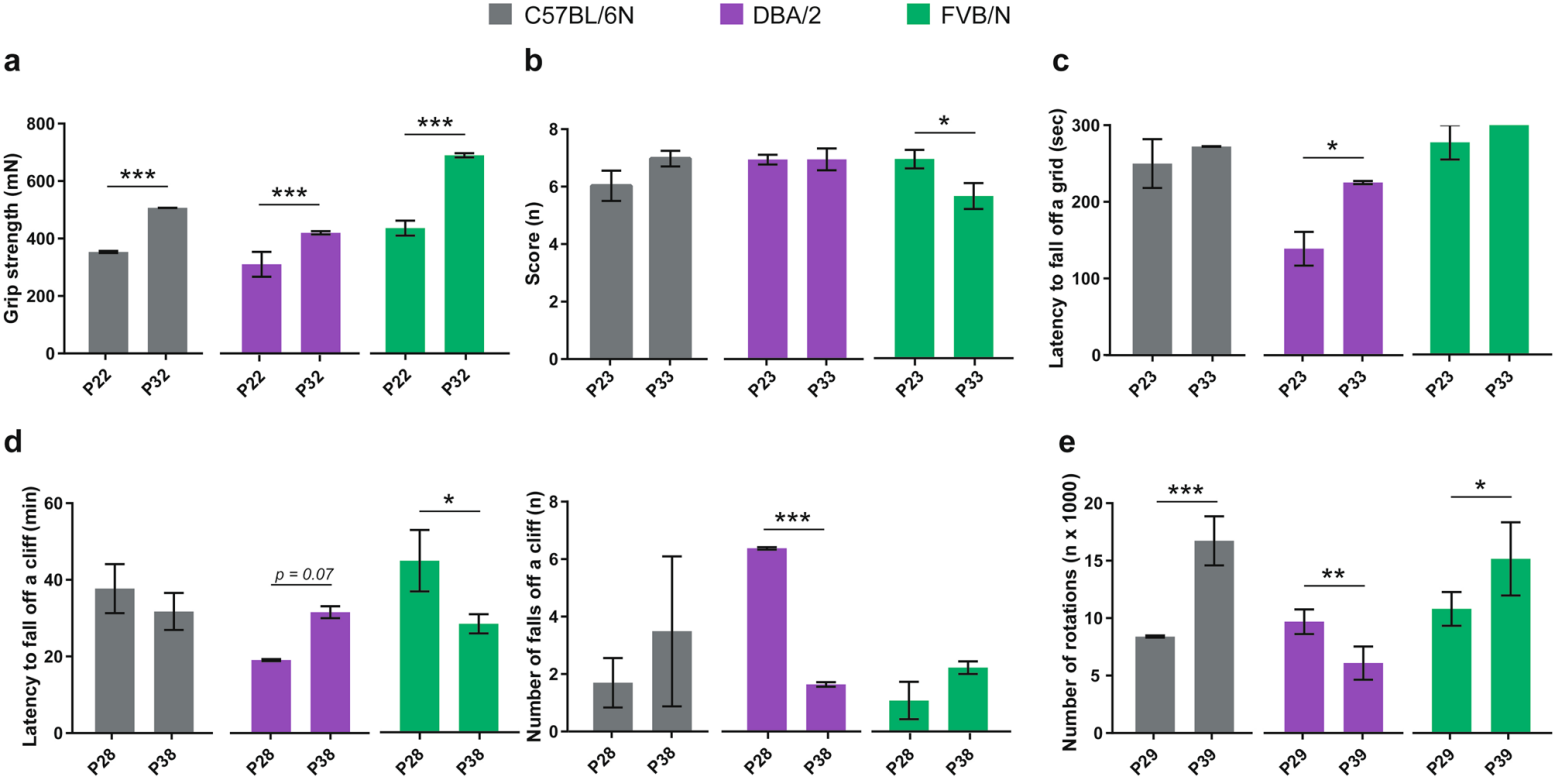
Comparison between the P22–30 and P32–40 cohorts within C57BL/6N, DBA/2, and FVB/N strains. (**a**) The grip strength was increased with age in the three investigated strains. (**b**) In the beam balance rod test, only FVB/N mice showed a decreased score in older mice. (**c**) The latency to fall off a grid in the inverted screen test was increased in older DBA/2 mice. (**d**) In the cliff avoidance reaction test, FVB/N mice exhibited lower latency to fall off a cliff (left) and DBA/2 showed a decreased number of falls (right). **e)** The voluntary wheel running test revealed inconsistent results in different strains with C57BL/6N and FVB/N mice showing an increase in the number of rotations with age in contrast to DBA/2 mice. Two-way ANOVA followed by Tukey post hoc test,*p ≤ 0.05, **p ≤ 0.01, ***p ≤ 0.001. Error bars indicate the standard error of the mean (SEM).

To test whether the age difference of 10 days can affect the motor and coordination functions during adolescence, results of the behavioral test battery were compared between the two cohorts. In the grip strength test, all of the three investigated strains displayed increased grip strength with age (Fig. 5a). In contrast, other tests revealed inconsistent results between strains with FVB/N exhibiting a lower score in the beam balance rod test (Fig. 5b) and DBA/2 showing increased latency to fall off a grid in the inverted screen test in older mice (Fig. 5c). In the cliff avoidance reaction test, the latency to fall off a cliff in FVB/N mice, and the number of falls in DBA/2 mice were decreased with age (Fig. 5d). Interestingly, in the voluntary wheel running test, the number of rotations of C57BL/6N and FVB/N mice was increased with age in contrast to the number of rotations of DBA/2 mice which was decreased in older mice (Fig. 5e).

### Correlation between the weight of mice and performance in the motor and coordination tests

To test whether the weight of mice can be a factor in determining the performance on various motor and coordination tests, we calculated the Pearson's correlation coefficient for each test in the behavioral test battery for the P32–40 cohort. Only the grip strength and inverted screen tests were positively correlated with the weight of mice (Grip strength: Pearson’s *r* = 0.7068, *p* < 0.0001; Inverted screen: Pearson’s *r* = 0.4543, *p* = 0.0008; Beam balance rod test: Pearson’s *r* = − 0.1476, *p* = 0.303306; Latency to fall in the cliff avoidance: Pearson’s *r* = 0.0394, *p* = 0.783698; Number of falls in the cliff avoidance: Pearson’s *r* = − 0.2059, *p* = 0.148999; Voluntary wheel running: Pearson’s *r* = 0.1292, *p* = 0.366211). These results indicate that the increased body weight of mice can increase the grip strength and thus, the latency to fall off a grid.

## Discussion

Studying the behavior of genetically-modified rodents modeling neuropsychiatric disorders contributes to our understanding of the pathophysiology of these disorders and enhances the development of new therapeutics. Therefore, it is important to perform behavioral experiments during the developmental time window that matches the onset of symptoms in humans before or around puberty corresponding to P42 in mice^50,51^. In our previous studies, we showed that several experiments assessing different behavioral domains related to neuropsychiatric disorders can be performed during adolescence^42,43^. These tests included USV recording, LABORAS, anxiety-related tests (Open field, elevated plus maze, dark/light compartment, and hole-board tests), and cognitive tests (Puzzle box, fear conditioning, and active place avoidance tests). As motor abnormalities in neuropsychiatric disorders appear even before the onset of the core symptoms, it becomes mandatory to assess motor functions in young mice. Moreover, impaired motor functions can influence findings in other tasks, such as cognitive tests.

We here investigated which behavioral experiments assessing motor activity and coordination function can be performed during adolescence and established baseline levels of motor ability for three inbred wild-type mouse strains commonly used in biomedical and behavioral research. Our test battery covered the most frequently used assays including the grip strength, beam balance rod, inverted screen, cliff avoidance reaction, rotarod, and voluntary wheel running tests. To our knowledge, this is the first comprehensive study performed in young mice thoroughly exploring motor functions in different inbred strains. All three strains showed the ability to perform these tests in two developmental stages of adolescence. However, the three strains differed, with DBA/2 mice displaying weaker motor and coordination functions. These strain variations stress that the results of motor functions should be included to correctly interpret the outcome of other behavioral tests.

The rotarod test is one of the oldest tests that has been extensively used to assess the innate motor ability in mice since 1950s^52,53^. Moreover, by performing several trials over multiple days, the rotarod test can unravel the motor learning ability that can indicate the degree of synaptic plasticity in various mouse strains during different developmental stages. Three studies comprehensively compared the performance of several inbred mouse strains on the rotarod. In the first study by Tarantino et al. (2000), four mouse strains, C57BL/6J, C3HeB/FeJ, DBA/2J, and 129/SvImJ, and aged around P60 were compared in an accelerating rotarod paradigm with three trials per day for two consecutive days^54^. Consistent with our finding, C57BL/6J mice stayed longer on the rotarod than DBA/2J mice. In the second study, the ability of eight inbred strains to perform on the rotarod was investigated with nine trials across three days^55^. In that study, C57BL/6J mice exhibited better performance on the rotarod compared to DBA/2J and FVB/NJ mice consistent with our findings^55^. When comparing the two previously mentioned studies together, DBA/2J mice performed better and showed increased motor learning in Tarantino et al.^54^ study than in McFadyen et al.^55^, which can be explained by the different protocols used in both studies. In the third study in young mice aged 5–6 weeks, again consistent with our results, C57BL/6J mice had the highest latencies for remaining on the rotarod compared to DBA/2J and FVB/NJ^56^. However, these results were in contrast to a previous study by the same group showing no difference between C57BL/6J, DBA/2J, and FVB/NJ mice^57^. On the other hand, motor and coordination functions differed between substrains. C57BL/6J mice exhibited significantly longer latencies than C57BL/6N and C57BL/6C mice^58^. Thus, care needs to be taken when comparing the results of studies in different mouse substrains. Notably, in our study, several mice made passive rotations i.e., mice held onto the bar and rotated for at least one revolution. If a mouse clings onto the rod in the rotarod test instead of running on top of it, no conclusions about its balance and coordination can be made^59,60^. Accordingly, we considered the first passive rotation of a mouse as the end of the trial since it can indicate the lost capability in the rotarod performance. As some laboratories consider the fall of the mouse off the rotarod as an endpoint, the different evaluation protocols will, at least partly, account for the lack of inter-laboratory reproducibility. Additionally, the apparatus itself including the diameter of the rod, material covering the rod, speed, and rate of acceleration of the rod can also impact the variability of behavioral results between laboratories^59,61,62^.

Surprisingly, different coordination tests revealed intrastrain diverging results. For example, the P22–30 DBA/2 mice showed less coordination in the inverted screen and cliff avoidance tests but not the beam balance rod test. This implies that several behavioral tests assessing motor activity and coordination function need to be approached for a full characterization of genetically-engineered mice modeling neuropsychiatric disorders.

Measuring motor function in both male and female mice is of particular relevance as the susceptibility to neuropsychiatric disorders differs between males and females with a ratio of 4:1 in ASD^63–66^, 1.4:1 in schizophrenia^67,68^, 4:1 in attention-deficit hyperactivity disorder^69–72^ and 1:2 in depression^73–78^. Additionally, neuropsychiatric disorders exhibit sex differences in the age of onset, disease progression, and disease severity^79,80^. Females are known to reach puberty, the major feature of adolescence^81^, before males, which can have an impact on the behavior during this sensitive period. Various markers are used to determine puberty in mice including the vaginal opening, first vaginal cornification, onset of cyclicity in females and balanopreputial separation in males. These different markers make it difficult to determine the precise date of puberty in mice. Additionally, differences in the onset of puberty have been reported in different inbred strains, which add another layer of complexity in comparing the behavioral results of different strains. For instance, the vaginal opening in female DBA/2J and C57BL/6J mice occur, on average, at P25.78 and P26.45, respectively^82^. In contrast, the onset of cyclicity was earlier in female C57BL/6J (P48.67) than DBA/2J (P51.11) mice. For males, the occurrence of the balanopreputial separation was earlier in C57BL/6J (P29.71) than DBA/2J (P34) mice^82^. To this end, a rough dating of puberty in conventional preclinical models using laboratory wild-type and genetically modified mice is recommended for the accurate characterization of the behavioral outcome.

In our study, the C57BL/6N strain showed in the rotarod test the strongest difference between male and female mice with better performance by the latter. The difference being stronger in P26–27 than P36–37 mice may indicate an interrelation between age and sex. The better performance of female than male C57BL/6N mice is consistent with two previous findings in C57BL/6J mice tested at different ages^55,83^. In P60–90 mice, female C57BL/6J mice had longer latency to fall off the rotarod than males^55^. In another study, female P30 C57BL/6J mice showed a trend towards increased duration on the rotarod, which reached significance at P150^83^, again confirming the effect of age on distinct behaviors. In contrast to C57BL/6N, DBA/2 mice in our study revealed no difference between males and females in both cohorts in all tests except the grip strength at P22. These results are consistent with a study showing no sex difference in DBA/2 mice in the rotarod test^55^ and with our previous study revealing no sex differences in DBA/2 mice in other behavioral tests related to neuropsychiatric disorders^43^. Furthermore, female FVB/N mice revealed more activity than males on the running wheel at P39, which is in line with our previous results showing a higher activity of P36 females in the home cage LABORAS test^43^. In summary, the sex-specific differences in motor activity and coordination function stress the necessity of testing both males and females in phenotyping mouse models of neuropsychiatric disorders.

The 10-day interval between the two tested cohorts displayed strong differences in the behavioral outcome, mainly depending on the investigated strain and behavioral paradigm. Indeed, several studies revealed aging from young to middle age inducing not only increased body weight^84^ but also reduced rotarod performance^85,86^, and altered locomotor activity^87^. Our results highlight the importance of small developmental windows and stress the importance of comparing mice of the same age since a few days’ differences can have a strong impact on the behavioral pattern.

The positive correlation between the weight of the mouse and performance on the grip strength and inverted screen tests needs to be taken into consideration when characterizing motor functions. Taking the weight into account can enhance the reproducibility of behavioral tests in genetically-modified mouse models, a main issue in the behavioral neuroscience field^88^. Consistent with a previous study performed on P30 and P150 C57BL/6J mice^83^, our analysis of male and female C57BL/6N mice revealed no statistically significant correlation between the body weight and rotarod performance. In contrast, a significant negative correlation was found in P60–90 C57BL/6J mice^55^. The discrepancy of the results in the aforementioned studies likely relies on age differences.

As young mice are more active and hectic than adult mice, the animal-experimenter interaction can have an impact on the behavioral results. Because high standardization facilitates inter-laboratory reproducibility, increases efficiency, and reduces variability^89^, many automated methods with minimum handling of mice such as automated home-cage wheel-running^90^, CatWalk gait analysis^91^, and Erasmus ladder test^92^, are meanwhile replacing manual ones. These tests with a little interaction with mice will allow more precise characterization of motor function, coordination, and motor learning. However, careful attention to other confounding variables during testing, statistical analysis and interpretation remain important and require consideration. In addition to the experimenter and way of handling^93,94^, a large array of factors can impact the behavioral outcome of rodents. Some published examples are the cage type, density and its type of enrichment^93,95–97^, and even the cage position on the rack in the vivarium^98^ can significantly alter rodent behavior. Geographical location also can affected body weight, motor coordination, and motor learning capability of wild-type mice, which partially relied on the rodent diet and water quality^83^. Therefore, all these factors must be ruled out for approaching genuine differences in motor function and learning.

In brief, differences in motor function as determined by performance in different motor and coordination tests uncovered the genetic difference as an important determinant in motor performance. Moreover, our study paves the way for better reproducibility of behavioral tests by addressing the effect of age during adolescence, sex, and weight on achieving the face validity of neuropsychiatric disorder-associated motor dysfunctions. Accordingly, behavioral characterization of knockout/knockin mice are recommended having littermates of the same delivery day, alike male/female ratios, and weight should be checked before and after every motor test since these three factors can strongly shift the results. Keeping these factors in mind will decrease variability, increase the face validity of the rodent model and concomitantly allow better defining “disease progression” and drug efficacy in motor abnormalities.

## Materials and methods

### Animals and housing conditions

Animals and housing conditions were similar to our previous study^43^. All procedures were conducted in strict compliance with national and international guidelines for the Care and Use of Laboratory Animals. The experiments on mice were approved by the local government (Regierungspräsidium Karlsruhe, Germany G-102/16) and were carried out in compliance with the ARRIVE guidelines.

### Experimental design and groups

All behavioral tests were carried out during the daylight cycle. Mice were brought into the behavioral room half an hour before the behavioral testing. Behavioral tests started at 7 am. We analyzed the behavior in 2 cohorts of group-housed mice of both sexes with one cohort starting at P22 till P30 and the second starting at P32 till P40. The number of mice per cohort and the type of the behavioral experiments are listed (Table 1). One male DBA/2 mouse died during the testing procedure and was not tested in the cliff avoidance reaction or voluntary wheel running tests in the P22–P30 cohort.

**Table 1:** Mouse cohorts, number and age of the adolescent mice used in the different experiments.

Cohorts	Strains	Mice (#)		Number of litters	Behavioral test at postnatal day (P#)
		Male ♂	Female♀		
P22–30					Grip strength (P22)
	C57BL/6N	6	9	2	Beam balance rod test/Inverted screen test (P23)
	DBA/2	13	9	3	Rotarod (P26–27)
	FVB/N	11	7	3	Cliff avoidance reaction test (P28)
					Voluntary wheel running activity (P29–30)
P32–40					Grip strength (P32)
	C57BL/6N	8	10	2	Beam balance rod test/Inverted screen test (P33)
	DBA/2	9	7	3	Rotarod (P36–37)
	FVB/N	8	9	2	Cliff avoidance reaction test (P38)
					Voluntary wheel running activity (P39–40)

The grip strength test was the first test to be applied to each cohort. One day later, we applied the beam balance rod test in the morning and the inverted screen test in the afternoon. Animals were maintained in the test room during this period. Then, we left the mice for two days without any experiments. At P26 and P36, we performed the rotarod test as one training trial permitting the mice to be habituated to the apparatus and to learn the task and two additional trials on the same day. On the following day, the data of three additional trials were collected. At P28 and P38, the cliff avoidance reaction test was performed. At P29 and P39, the voluntary wheel running test was performed for 24 h. The weights of male and female mice were measured before performing the tests and are shown in Supplementary Fig. 1 and Supplementary Table 1.

After each trial, the apparatuses were carefully cleaned with 75% ethanol solution wetted tissue paper. All behavioral experiments were performed in a randomized manner with a separation of experiment conduction and data analysis.

### The behavioral test battery

#### The grip strength test

The strength of the forelimb, as well as the overall neuromuscular strength, were tested using a grip strength meter (Ugo Basile, Gemonio, Italy, model 47106), which automatically measured the force (mN) needed for the mouse to release a grip after having to grasp and cling to it. During this task, the mouse was held by the tail. After an initial training session, the mice were tested for one session. A test session is consisting of three consecutive trials and the final result was calculated as the average of these three trials.

#### The beam balance rod test

The time during which the mouse was able to remain on a narrow, elevated rod without falling was measured. The apparatus is composed of 7 rods with diameters of 6, 8, 12, 15, 20, 25, and 32 mm, respectively. The length of each rod was 50 cm with a height of 20 cm above the ground. The mouse was put on each rod for 10 s starting from the widest to the narrowest. The score was calculated by the number of rods from which the tested mouse did not fall. If the mouse did not fall, the score was considered 8. The test was performed twice with 1 h interval.

#### The inverted screen test

Muscle strength using all four limbs was tested in a modified version of the inverted screen test. The mouse was placed in the center of a metal grid screen. The grip screen is then carefully rotated to an inverted position and held steadily 45 cm above an empty cage containing bedding. The latency to fall off the grid was recorded with a maximum trial time of 5 min.

#### The rotarod test

The accelerated rotarod (Ugo Basile, Gemonio, Italy, model 47600) test is a standard sensory-motor test to investigate animals’ motor coordination and learning skills by measuring the ability of the mouse to stay and run on the accelerated rod. The test was performed for a duration of 8 min with an acceleration of 4–40 rpm. As soon as a mouse fell off the rod or started to rotate with the rotarod without running, the time was stopped. After an initial training trial, mice were tested for five trials over two days. The recovery phase between trials was 10 min.

#### The cliff avoidance reaction test

The cliff avoidance reaction assesses coordination and maladaptive impulsive behavior. It was assessed using a round plastic platform (diameter, 20 cm) supported by a rod (height 50 cm) similar to a bar stool^99^. The floor below the platform was carpeted with smooth tissue to prevent injury if the animal fell. The test was initiated by gently placing the animal on the middle of the platform. The latency from an initial placement on the platform until falling was recorded. Mice that fell off the platform were immediately put back on the platform and the total number of falls during the entire session (60 min) was counted.

#### The voluntary wheel running test

The voluntary wheel running test was modified from^100^. Mice were placed individually in cages containing a running wheel and free access to food and water. The unrestricted voluntary wheel running activity started at 7–8 am and was digitally recorded for 24 h using the AWM counter (Lafayette Instrument, Louisiana, USA) that uses an optical sensor to detect the total revolutions of the wheel and is connected to a USB Interface and PC running an AWM Software (Lafayette Instrument, Louisiana, USA).

### Statistical analysis

Two-way ANOVA was used with sex and genotype as the two factors. This was followed by Tukey’s post hoc test for multiple comparisons to determine differences between the three strains C57BL/6N, DBA/2, and FVB/N and Bonferroni correction to check differences between males and females within each strain. To compare the two developmental stages (P22–30) and (P32–40) within each strain, two-way ANOVA was used with sex and age as the two factors. Additionally, a correlation analysis between the weight of P32–40 mice and their performance on the tests was done and is discussed in the results section. All data were expressed as mean ± SEM. A *P* value ≤ 0.05 was considered statistically significant. Statistical analysis was performed using GraphPad Prism 7 and Microsoft Office Excel software. The respective numbers of male and female mice are described in Table 1 and the individual figures.

## Supplementary Information

Supplementary Figures and Tables
