# Supplementary material for: Comprehensive characterization of motor and coordination functions in three adolescent wild-type mouse strains
Source: Sci Rep. 2021 Mar 22;11:6497. doi: 10.1038/s41598-021-85858-3 (PMC7985312; doi:10.1038/s41598-021-85858-3)
Supplement: Supplementary file 1 — Supplementary Figures and Tables [file 41598_2021_85858_MOESM1_ESM.docx]

**Comprehensive characterization of motor and coordination functions in three adolescent wild-type mouse strains**

Ahmed Eltokhi^1,2^*, Barbara Kurpiers^2^, Claudia Pitzer^2^*

^1^ Department of Neurology and Epileptology, Hertie Institute for Clinical Brain Research, University of Tübingen, Tübingen, Germany

^2^ Interdisciplinary Neurobehavioral Core, Heidelberg University, Heidelberg, Germany

The current address of Dr. Ahmed Eltokhi is: Department of Pharmacology, University of Washington, Seattle, USA

*Correspondence: Dr. Ahmed Eltokhi: Eltokhi@uw.edu, Dr. Claudia Pitzer: Claudia.pitzer@pharma.uni-heidelberg.de


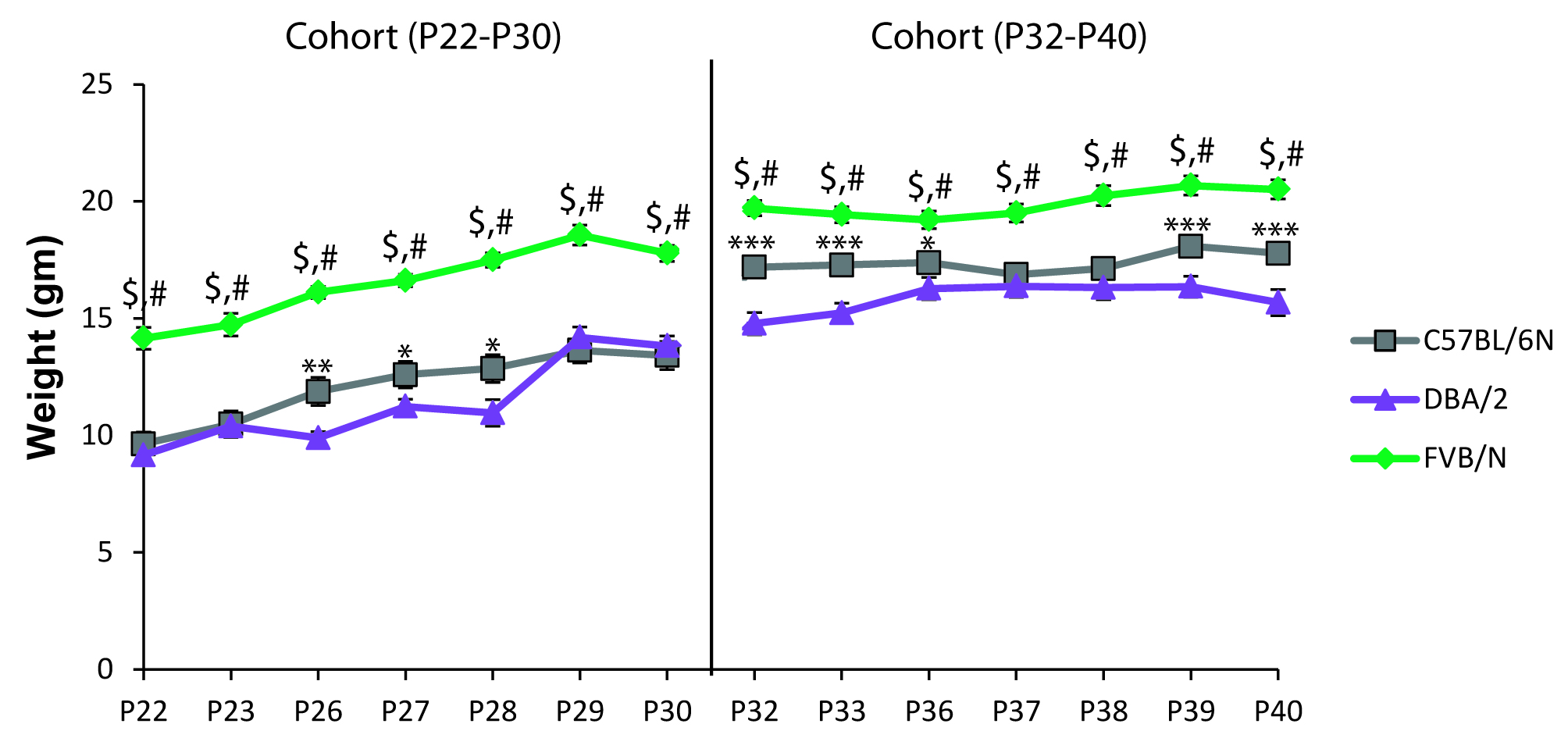


**Supplementary Figure 1: The weights of adolescent C57BL/6N, DBA/2, and FVB/N mice before each test in the behavioral test battery.** The tests are grip strength (at P22 and P32), beam balance rod and inverted screen (at P23 and P33), rotarod (at P26-27 and P36-37), cliff avoidance reaction (at P28 and P38), and voluntary wheel running (at P29-30 and P39-40). Two-way ANOVA followed by Tukey post hoc test, $ *p* ≤ 0.001 for FVB/N vs C57BL/6N; # *p* ≤ 0.001 for FVB/N vs DBA/2; **p* ≤ 0.05 and ***p* ≤ 0.01 for C57BL/6N vs DBA/2. Error bars indicate the standard error of the mean (SEM).


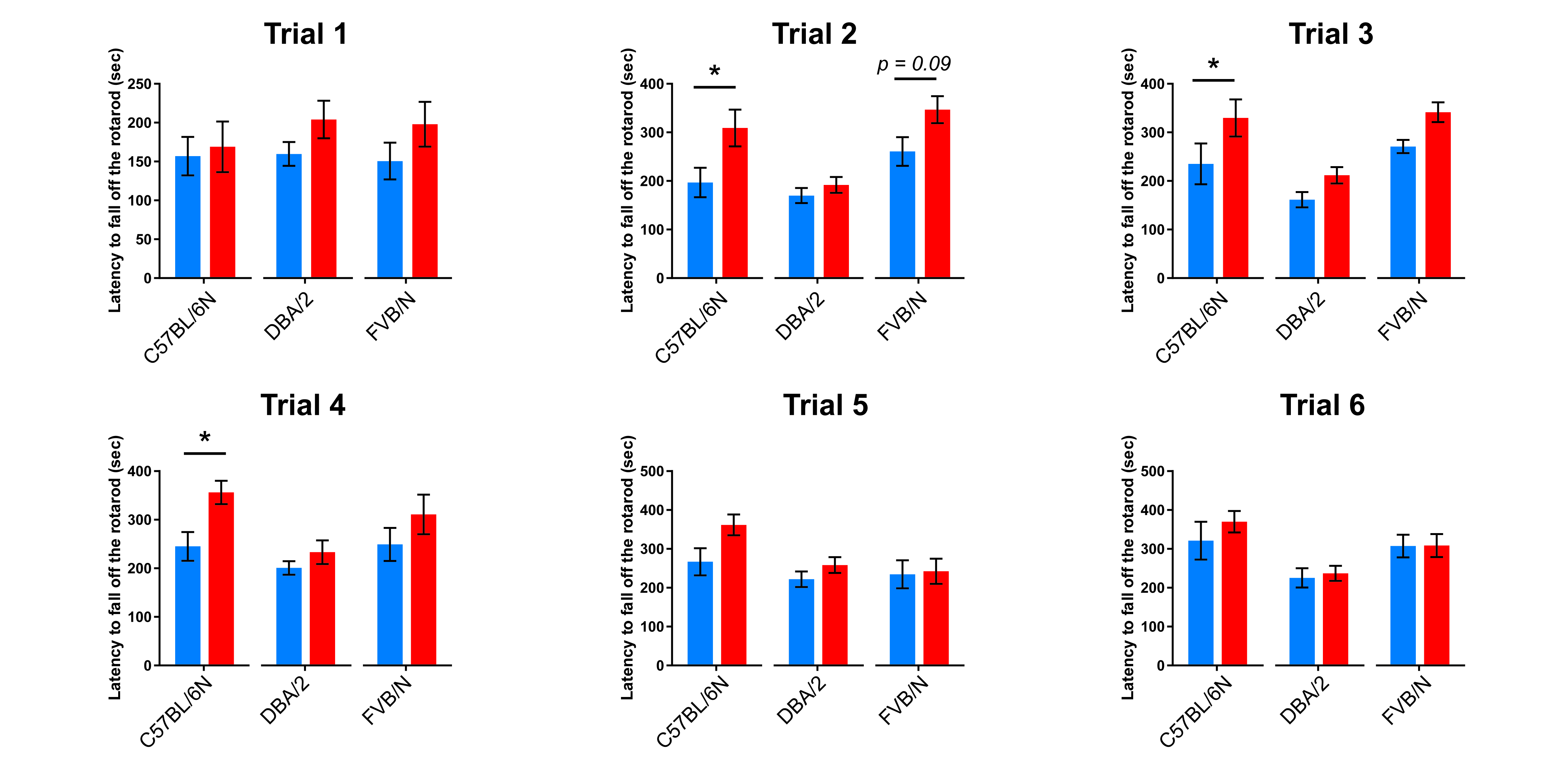


**Supplementary Figure 2:** **Comparison between male and female P26-27 mice of C57BL/6N, DBA/2, and FVB/N strains in the rotarod test.** Two-way ANOVA followed by Bonferroni post hoc test, **p* ≤ 0.05. Blue and red bars refer to males and females, respectively. Error bars indicate the standard error of the mean (SEM). For the detailed *p* values, see **Supplementary Table 2**.


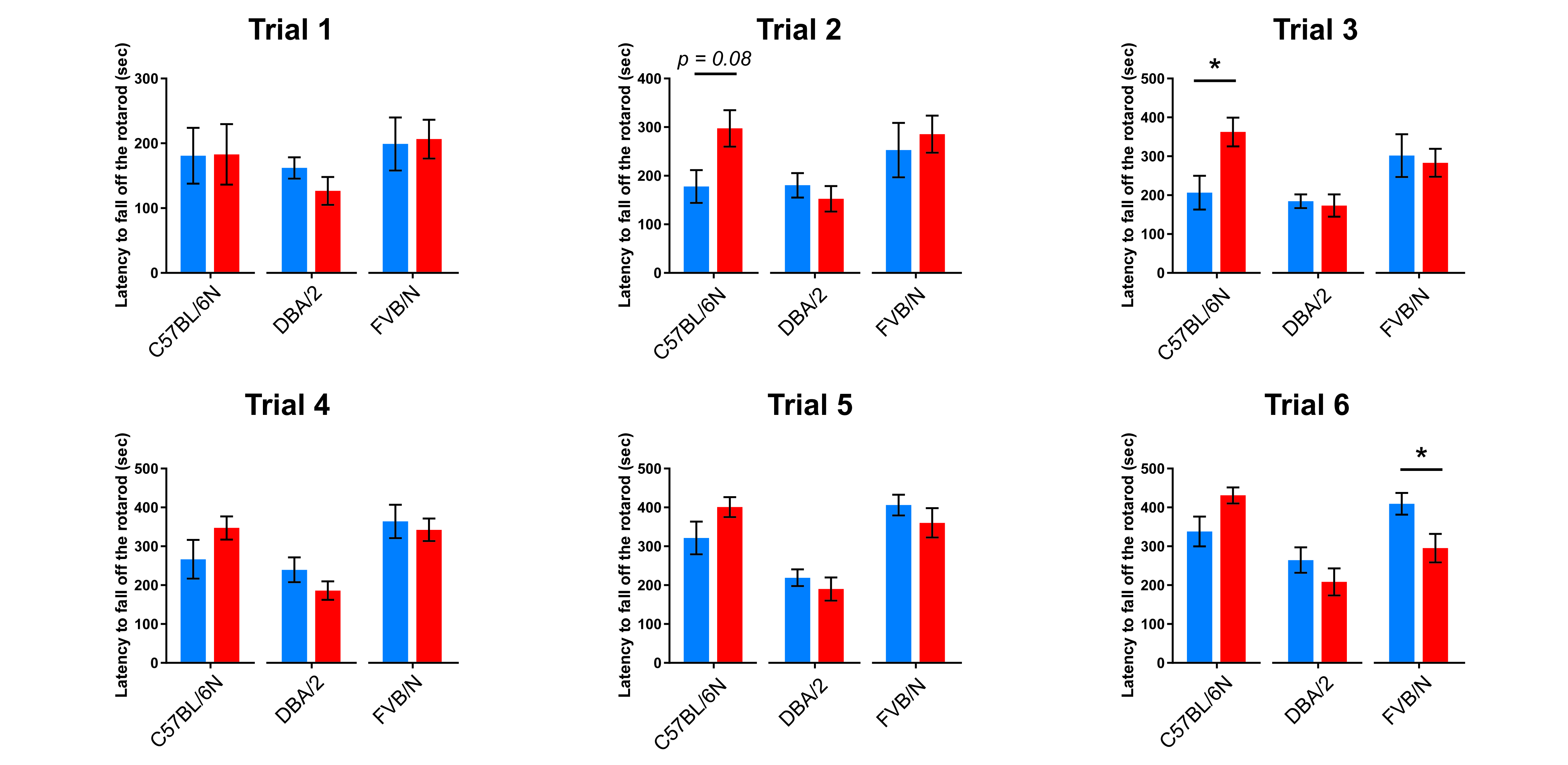


**Supplementary Figure 3: Comparison between male and female P36-37 mice of C57BL/6N, DBA/2, and FVB/N strains in the rotarod test.** Two-way ANOVA followed by Bonferroni post hoc test, **p* ≤ 0.05. Blue and red bars refer to males and females, respectively. Error bars indicate the standard error of the mean (SEM). For the detailed *p* values, see **Supplementary Table 2**.

**Supplementary Table 1: Weight comparison between male and female mice within each strain of C57BL/6N, DBA/2, and FVB/N.** The yellow highlights indicate significant results.

| **Name of test/age** | **C57BL/6N** |  |  | **DBA/2** |  |  | **FVB/N** |  |  |
| --- | --- | --- | --- | --- | --- | --- | --- | --- | --- |
|  | ♂ Mean +/- SEM | ♀ Mean +/- SEM | Bonferroni test | ♂Mean +/- SEM | ♀ Mean +/- SEM | Bonferroni test | ♂Mean +/- SEM | ♀ Mean +/- SEM | Bonferroni test |
| **Grip strength** |  |  |  |  |  |  |  |  |  |
| P22 | 9.40  +/-  1.13 | 9.80  +/-  0.46 | >0.9999 | 8.67  +/-  0.70 | 9.87  +/-  0.32 | 0.5446 | 14.12  +/-  0.57 | 14.21  +/-  0.87 | >0.9999 |
| P32 | 18.53  +/-  0.40 | 16.12  +/-  0.31 | 0.0005 | 15.71  +/-  0.48 | 13.57  +/-  0.67 | 0.0039 | 20.65  +/-  0.36 | 18.88  +/-  0.34 | 0.0152 |
| **Beam balance test and Inverted screen test** |  |  |  |  |  |  |  |  |  |
| P23 | 10.22  +/-  1.30 | 10.66  +/-  0.46 | >0.9999 | 10.25  +/-  0.30 | 10.60  +/-  0.27 | >0.9999 | 14.87  +/-  0.63 | 14.50  +/-  0.81 | >0.9999 |
| P33 | 18.58  +/-  0.33 | 16.24  +/-  0.28 | 0.0003 | 16.03  +/-  0.42 | 14.20  +/-  0.64 | 0.0077 | 20.44  +/-  0.32 | 18.53  +/-  0.37 | 0.0038 |
| **Rotarod** |  |  |  |  |  |  |  |  |  |
| P26 | 11.77  +/-  1.43 | 11.96  +/-  0.44 | >0.9999 | 9.71  +/-  0.40 | 10.17  +/-  0.27 | >0.9999 | 16.41  +/-  0.37 | 15.66  +/-  0.41 | 0.9933 |
| P27 | 12.60  +/-  1.38 | 12.59  +/-  0.36 | >0.9999 | 11.16  +/-  0.48 | 11.33  +/-  0.77 | >0.9999 | 17.05  +/-  0.29 | 15.94  +/-  0.39 | 0.4858 |
| P36 | 18.71  +/-  0.34 | 16.32  +/-  0.26 | 0.0003 | 17.27  +/-  0.45 | 15.00  +/-  0.66 | 0.0014 | 20.39  +/-  0.39 | 18.17  +/-  0.38 | 0.0012 |
| P37 | 18.41  +/-  0.39 | 15.64  +/-  0.24 | <0.0001 | 17.38  +/-  0.47 | 15.09  +/-  0.67 | 0.0010 | 20.79  +/-  0.37 | 18.36  +/-  0.34 | 0.0003 |
| **Cliff avoidance test** |  |  |  |  |  |  |  |  |  |
| P28 | 13.05  +/-  1.41 | 12.73  +/-  0.42 | >0.9999 | 11.33  +/-  0.51 | 10.48  +/-  1.18 | >0.9999 | 18.00  +/-  0.33 | 16.69  +/-  0.476 | 0.6588 |
| P38 | 18.54  +/-  0.35 | 16.00  +/-  0.19 | 0.0003 | 17.39  +/-  0.56 | 14.93  +/-  0.61 | 0.0011 | 21.55  +/-  0.51 | 19.08  +/-  0.35 | 0.0007 |
| **Voluntary wheel running activity** |  |  |  |  |  |  |  |  |  |
| P29 | 13.65  +/-  1.33 | 13.62  +/-  0.31 | >0.9999 | 14.51  +/-  0.69 | 13.77  +/-  0.46 | >0.9999 | 19.43  +/-  0.46 | 17.20  +/-  0.51 | 0.0571 |
| P30 | 13.37  +/-  1.47 | 13.46  +/-  0.38 | >0.9999 | 14.15  +/-  0.68 | 13.38  +/-  0.41 | >0.9999 | 18.35  +/-  0.39 | 16.89  +/-  0.48 | 0.3537 |
| P39 | 19.44  +/-  0.41 | 17.00  +/-  0.26 | 0.0002 | 17.39  +/-  0.45 | 15.03  +/-  0.54 | 0.0006 | 22.03  +/-  0.36 | 19.48  +/-  0.39 | 0.0001 |
| P40 | 19.13  +/-  0.38 | 16.71  +/-  0.20 | 0.0001 | 17.13  +/-  0.61 | 13.81  +/-  0.33 | <0.0001 | 21.93  +/-  0.35 | 19.24  +/-  0.34 | <0.0001 |

**Supplementary Table 2: Comparison between male and female mice of C57BL/6N, DBA/2, and FVB/N strains in the behavioral test battery.** The yellow highlights indicate significant results.

| **Name of test** | **C57BL/6N** |  |  | **DBA/2** |  |  | **FVB/N** |  |  |
| --- | --- | --- | --- | --- | --- | --- | --- | --- | --- |
|  | ♂ Mean +/- SEM | ♀ Mean +/- SEM | Bonferroni test | ♂Mean +/- SEM | ♀ Mean +/- SEM | Bonferroni test | ♂Mean +/- SEM | ♀ Mean +/- SEM | Bonferroni test |
| **Grip strength** |  |  |  |  |  |  |  |  |  |
| Grip strength (at P22) | 349.50  +/-  24.05 | 356.70  +/-  14.08 | >0.9999 | 267.03  +/-  18.68 | 353.81  +/-  13.64 | 0.0166 | 410.36  +/-  25.17 | 462.19  +/-  38.95 | 0.3792 |
| Grip strength (at P32) | 507.25  +/-  23.78 | 506.37  +/-  18.53 | >0.9999 | 425.78  +/-  20.31 | 414.19  +/-  23.27 | >0.9999 | 682.38  +/-  16.68 | 696.74  +/-  19.30 | >0.9999 |
| **Beam balance test** |  |  |  |  |  |  |  |  |  |
| Score (at P23) | 5.50  +/-  0.922 | 6.56  +/-  0.47 | 0.5677 | 6.77  +/-  0.46 | 7.11  +/-  0.26 | >0.9999 | 6.64  +/-  0.49 | 7.29  +/-  0.36 | >0.9999 |
| Score (at P33) | 7.25  +/-  0.31 | 6.70  +/-  0.21 | >0.9999 | 7.33  +/-  0.24 | 6.57  +/-  0.20 | 0.8541 | 6.13  +/-  0.66 | 5.22  +/-  0.81 | 0.5702 |
| **Inverted screen test** |  |  |  |  |  |  |  |  |  |
| Latency (at P23) | 218.00  +/-  52.23 | 281.89  +/-  18.11 | 0.7147 | 116.92  +/-  34.12 | 160.78  +/-  45.40 | 0.9723 | 255.27  +/-  25.16 | 300.00  +/-  0.00 | >0.9999 |
| Latency (at P33) | 272.63  +/-  27.38 | 271.80  +/-  28.20 | >0.9999 | 227.2222  +/-  39.13714 | 223.14  +/-  49.63 | >0.9999 | 300.00  +/-  0.00 | 300.00  +/-  0.00 | >0.9999 |
| **Rotarod** |  |  |  |  |  |  |  |  |  |
| Duration (Trial 1 at P26) | 157.00  +/-  24.73 | 169.00  +/-  32.54 | >0.9999 | 159.77  +/-  15.32 | 204.11  +/-  24.18 | 0.5231 | 150.55  +/-  23.63 | 198.00  +/-  28.83 | 0.5759 |
| Duration (Trial 2 at P26) | 196.83  +/-  30.45 | 309.00  +/-  37.94 | 0.0321 | 169.92  +/-  15.42 | 191.78  +/-  16.25 | >0.9999 | 260.73  +/-  29.44 | 346.86  +/-  27.73 | 0.0929 |
| Duration (Trial 3 at P26) | 235.17  +/-  42.07 | 329.67  +/-  38.17 | 0.0484 | 161.54  +/-  15.62 | 211.78  +/-  16.85 | 0.3412 | 270.90  +/-  13.43 | 341.57  +/-  20.28 | 0.1429 |
| Duration (Trial 4 at P27) | 245.00  +/-  29.49 | 356.22  +/-  24.04 | 0.0420 | 200.77  +/-  13.81 | 233.11  +/-  24.35 | >0.9999 | 249.18  +/-  33.87 | 310.86  +/-  40.69 | 0.3894 |
| Duration (Trial 5 at P27) | 266.67  +/-  34.76 | 361.56  +/-  26.95 | 0.1288 | 221.923  +/-  19.99 | 258.22  +/-  20.23 | >0.9999 | 234.45  +/-  36.06 | 242.14  +/-  32.49 | >0.9999 |
| Duration (Trial 6 at P27) | 321.17  +/-  48.83 | 369.78  +/-  27.48 | 0.8957 | 225.31  +/-  24.75 | 236.89  +/-  19.38 | >0.9999 | 307.27  +/-  29.00 | 308.57  +/-  29.50 | >0.9999 |
| Learning ratio (Trial 1 vs trial 6) | 2.41  +/-  0.65 | 3.03  +/-  0.65 | 0.9066 | 1.47  +/-  0.13 | 1.36  +/-  0.25 | >0.9999 | 2.33  +/-  0.25 | 1.79  +/-  0.34 | 0.9449 |
| Duration (Trial 1 at P36) | 180.88  +/-  42.96 | 182.90  +/-  46.61 | >0.9999 | 162.00  +/-  16.32 | 126.71  +/-  21.47 | >0.9999 | 199.00  +/-  40.84 | 206.56  +/-  29.91 | >0.9999 |
| Duration (Trial 2 at P36) | 177.75  +/-  33.68 | 297.40  +/-  37.55 | 0.0800 | 180.33  +/-  25.26 | 152.43  +/-  26.32 | >0.9999 | 252.88  +/-  55.91 | 285.56  +/-  38.16 | >0.9999 |
| Duration (Trial 3 at P36) | 206.50  +/-  43.23 | 362.50  +/-  36.82 | 0.0137 | 184.44  +/-  17.41 | 173.29  +/-  28.59 | >0.9999 | 302.00  +/-  54.99 | 283.22  +/-  36.08 | >0.9999 |
| Duration (Trial 4 at P37) | 266.63  +/-  49.89 | 347.10  +/-  29.80 | 0.3228 | 239.56  +/-  31.81 | 185.86  +/-  23.76 | 0.9240 | 364.00  +/-  43.08 | 342.44  +/-  29.05 | >0.9999 |
| Duration (Trial 5 at P37) | 321.38  +/-  42.10 | 400.90  +/-  25.57 | 0.2155 | 219.11  +/-  21.51 | 190.00  +/-  29.94 | >0.9999 | 406.00  +/-  26.76 | 360.33  +/-  37.82 | 0.9207 |
| Duration (Trial 6 at P37) | 338.00  +/-  38.41 | 430.90  +/-  20.62 | 0.1229 | 264.44  +/-  32.73 | 208.43  +/-  34.90 | 0.7158 | 409.38  +/-  27.81 | 295.11  +/-  36.76 | 0.0453 |
| Learning ratio (Trial 1 vs trial 6) | 2.60  +/-  0.55 | 3.84  +/-  0.82 | 0.3566 | 1.75  +/-  0.24 | 2.04  +/-  0.45 | >0.9999 | 2.67  +/-  0.49 | 1.84  +/-  0.48 | 0.9068 |
| **Cliff avoidance test** |  |  |  |  |  |  |  |  |  |
| Latency (at P28) | 44.10  +/-  7.87 | 31.31  +/-  7.50 | 0.6352 | 18.78  +/-  3.40 | 19.35  +/-  7.88 | >0.9999 | 36.96  +/-  5.79 | 53.01  +/-  6.99 | 0.2694 |
| Number of falls (at P28) | 0.83  +/-  0.48 | 2.56  +/-  1.82 | >0.9999 | 6.42  +/-  2.30 | 6.33  +/-  2.50 | >0.9999 | 1.73  +/-  0.41 | 0.43  +/-  0.43 | >0.9999 |
| Latency (at P38) | 36.56  +/-  7.17 | 26.92  +/-  6.75 | >0.9999 | 33.10  +/-  7.81 | 29.96  +/-  7.95 | >0.9999 | 26.05  +/-  7.86 | 31.02  +/-  7.53 | >0.9999 |
| Number of falls (at P38) | 0.88  +/-  0.30 | 6.10  +/-  2.08 | 0.0072 | 1.56  +/-  0.50 | 1.71  +/-  0.64 | >0.9999 | 2.00  +/-  0.63 | 2.44  +/-  1.03 | >0.9999 |
| **Voluntary wheel running activity** |  |  |  |  |  |  |  |  |  |
| Number of rotations (at P29-P30) | 8310.33  +/-  1519.93 | 8487.56  +/-  727.90 | >0.9999 | 8631.58  +/-  638.92 | 10759.33  +/-  1504.11 | 0.5796 | 12268.73  +/-  1121.49 | 9341.29  +/-  2039.00 | 0.3127 |
| Number of rotations (at P39-P40) | 14585.50  +/-  1522.13 | 18855.80  +/-  1871.19 | 0.1774 | 4640.44  +/-  875.6504 | 7537.86  +/-  1504.36 | 0.6681 | 11978.75  +/-  1854.67 | 18344.44  +/-  1603.64 | 0.0215 |
